# Attitudes toward communication skills with learner needs assessment within radiology residency programs in China: a cross-sectional survey

**DOI:** 10.1186/s13104-024-06779-8

**Published:** 2024-04-23

**Authors:** GengPeng Lian, Yubin Xiao, Yingling Huang, Huanpeng Wang, Lipeng Huang, Hongwu Yang, Chunmin Zhu, Wei Mei, Ruibin Huang

**Affiliations:** Department of Radiology, First Affiliated Hospital, Shantou University Medical College, Shantou, 515041 Guangdong China

**Keywords:** Radiology residents, Standardized residency training, Communication skills, Medical education, Needs assessment

## Abstract

**Background:**

Communication skills (CS) represent a core competency in radiology residency training. However, no structured curriculum exists to train radiology residents in CS in China. The aim of this study was to evaluate the status and prevalence of doctor–patient communication training among radiology residents in nine Chinese accredited radiology residency training programs and to determine whether there is a perceived need for a formalized curriculum in this field.

**Methods:**

We administered a cross-sectional online survey to radiology residents involved in CS training at nine standard residency training programs in China. The questionnaire developed for this study included CS training status, residents’ demographics, attitudes toward CS training, communication needs, and barriers. Residents’ attitudes toward CS training were measured with the Communication Skills Attitude Scale (CSAS) and its subscales, a positive attitude scale (PAS) and negative attitude scale (NAS).

**Results:**

A total of 133 (48.36%) residents participated in the survey. The mean total scores on the two dimensions of the CSAS were 47.61 ± 9.35 in the PAS and 36.34 ± 7.75 in the NAS. Factors found to be significantly associated with the PAS included receiving previous training in CS, medical ethics, or humanities and the doctor’s attire. We found that first-year residents and poor personal CS were the most influential factors on the NAS. Only 58.65% of participants reported having previously received CS training during medical school, and 72.93% of respondents reported failure in at least one difficult communication during their residency rotation. Most of those surveyed agreed that CS can be learned through courses and were interested in CS training. Some of the most common barriers to implementing formal CS training were a lack of time, no standardized curriculum, and a lack of materials and faculty expertise.

**Conclusions:**

Most residents had a very positive attitude toward CS training and would value further training, despite the limited formal CS training for radiology residents in China. Future efforts should be made to establish and promote a standard and targeted CS curriculum for Chinese radiology residents.

**Supplementary Information:**

The online version contains supplementary material available at 10.1186/s13104-024-06779-8.

## Background

Doctor–patient communication (DPC) is defined as a specific form of interpersonal communication that involves sharing information, listening attentively, building trust and respect, managing emotions, and sharing decision-making between providers and patients through language and behaviour [1]. Effective DPC is an essential aspect of quality patient care, ensuring patient compliance with physician recommendations, improving patient satisfaction, symptom resolution and treatment outcomes across many medical specialties, including radiology [2–4]. Whereas most medical imaging results have traditionally been sent directly to the referring physician, there is a growing emphasis on direct communication with the patient [5]. Recent studies have shown that patients have a preference for discussing their imaging results directly with radiologists [6, 7]. In addition to patient preference, previous studies have demonstrated the potential benefits to patient care, such as reducing errors, improving adherence to radiology recommendations and reducing delays to patient care [7–9].

Communication skills (CS) represent one of the core competencies in residency training [10–13]. The importance of training and regular assessment of residents’ CS has gained momentum [10–12, 14]. By 2013, China set the “5 + 3” rule that includes 3 years of residency training after the bachelor’s degree [15]. Unfortunately, according to a recent National Survey of Radiology Residency Training in China, the main focus of training programs is on patient care and medical knowledge, rather than on other “soft” competencies such as CS [16]. In particular, to our knowledge, no curriculum has been designed specifically for radiology residents, despite the standardized residency training (SRT) being in place since 2013.

The primary aim of the present survey was thus to determine the status and prevalence of DPC training among radiology residents in nine Chinese accredited radiology residency training programs. We also explored the perceptions, attitudes, and behaviors of radiology residents in relation to DPC and whether there is a perceived need for a formalized curriculum in this area.

## Methods

### Study design and participants

The study design was cross-sectional. To ensure that all survey questions were culturally and linguistically appropriate and easily understandable, the survey was first administered in a pilot study with 24 radiology residents from one institution, and all self-designed items and validated scales were modified accordingly. We applied a simple random sampling method to select nine radiology residency programs located in eastern, western, southern, northern, and central Guangdong Province, China. All these enrolled institutions are tertiary hospitals (hospital offering advanced specialized medical and health services to multiple regions) and SRT sites certified by the Guangdong Municipal Health Commission. Ethical approval for the study was given by the Ethics Commission of the First Affiliated Hospital of Shantou University Medical College (No. B2023020).

### Questionnaire

An interdisciplinary research group at The First Affiliated Hospital of Shantou University Medical College, including radiologists, medical communication experts, and psychologists who had been working for > 10 years, developed the questionnaire based on previous literature reviews, group discussion, and mock interviews. The questionnaire comprised binary response items (Yes/No), Likert-style questions, multiple-choice questions, and subjective responses. A detailed description of the questionnaire is provided in Additional file 1.

The questionnaire comprised four sections. The first part included items regarding sociodemographic data (age, sex, education, marital status, year of residency) and a self-assessment of CS. The second part included questions on the residents’ knowledge, experience, and confidence regarding DPC in radiology. This section started with the question, rating how stressful the current doctor–patient relationship is in general. Then, the participants were asked, if they had received any CS or medical ethics training in medical school. It has been reported that the doctor’s attire functions as a symbol of recognition, professionalism, and trust [17]. Thus, the participants were asked whether the white coat and the use of formal clothes could be an effective non-verbal communication tool to establish a good doctor–patient relationship. Subsequently, participants were asked whether they had had any difficult conversations (e.g., breaking bad news, disclosing medical errors, etc.) during their residency training. Moreover, the participants were asked about potential causing factors and the ways to resolve the failed DPC. Finally, participants rated whether the failure of the DPC harmed their clinical work using a 5-point Likert scale, with 1 being the most strongly disagree and 5 being the most strongly agree.

The third section of the questionnaire was based on the Communication Skills Attitude Scale (CSAS) [18], which is the most widely used tool for assessing students’ attitudes toward CS learning. We used the Chinese version, as translated previously [19]. The scale has two subscales with 13 items on each. Subscale I represents positive attitudes to CS learning (PAS), e.g. “Learning communication skills is interesting” (1 = strongly disagree to 5 = strongly agree), while Subscale II represents negative attitudes (NAS), e.g. “Communication skills training states the obvious and then complicates it” (1 = strongly agree, 5 = strongly disagree). The scores for each scale range from 13 to 65. The higher the overall score reached by a respondent, the stronger their positive or negative attitudes toward learning CS. The fourth part of the questionnaire queried regarding residents’ interest in receiving formal training in doctor–patient CS and barriers to implementation. A pilot study was conducted at the First Affiliated Hospital of Shantou University Medical College, and the questionnaire demonstrated good reliability and validity with Cronbach’s alpha of 0.84.

### Procedures

The survey was conducted using the electronic online survey tool “Questionnaires Star” (https://www.wjx.cn, China) during March 8–22, 2023. We asked the recruited radiology residents to scan the Quick Response (QR) code and enter the WeChat Mini Program (Questionnaires Star) to answer the questionnaire independently within the specified time. The researchers provided in-person technical assistance for barriers to using the online platform to control data quality. The submission of questionnaires could be checked on the platform. To minimize the sampling bias, we set the inclusion criterion for radiology residents as those who had teaching experience of more than half of a year. Participation was voluntary and consent to participate was included in the questionnaire.

### Statistical analysis

The data collected from the survey were statistically analyzed using IBM SPSS 25.0 (IBM Corp., Armonk, NY, USA). Categorical variables are reported as frequencies (percentage). Continuous variables with normal distributions are reported as mean ± standard deviation. The differences in NAS and PAS scores across categorical groups were analysed using independent samples t-test for two groups or one-way analysis of variance (ANOVA) for more than two groups. We considered a *p*-value of less than 0.05 to indicate statistical significance.

## Results

### Residents’ demographic characteristics

From the number of residents in the nine programs, the surveys were distributed to 275 residents. A total of 133 residents completed and returned the survey; therefore, the response rate was 48.36% (133/275). Among participants, 56.39% were women, 66.92% held a bachelor’s degree, and 85.71% were single or divorced. The average age was 26.4 years. The participants included 48 residents (36.09%) who were in their first year of residency, 35 residents (26.31%) who were in their second year, and 50 residents (37.60%) who were in their third year. The demographic characteristics of the participating residents and PAS and NAS scores according to demographic groups are shown in Table 1.

**Table 1:** Demographic characteristics of residents and PAS and NAS scores across categorical groups (n = 133)

Items	N	%	PAS			NAS		
			Mean (SD)	F/t	P value	Mean (SD)	F/t	P value
Gender								
Male	58	43.61	46.00 (10.09)	t = -1.76	0.08	37.03 (9.29)	t = 0.91	0.36
Female	75	56.39	48.85 (8.58)			35.80 (6.32)		
Marital Status								
Single/divorced	114	85.71	48.07 (8.53)	t = 1.39	0.16	36.20 (7.67)	t = -0.49	0.62
Married	19	14.29	44.84 (13.19)			37.15 (8.35)		
Education								
Bachelor’s degree	89	66.92	47.05 (9.77)	t = -0.97	0.33	36.96 (8.46)	t = 1.33	0.18
Master’s degree or above	44	33.08	48.72 (8.41)			35.06 (5.94)		
Resident grade								
I	48	36.09	47.35 (6.78)	F = 0.43	0.65	37.52 (6.99)	F = 3.36	0.03*
II	35	26.31	46.65 (10.23)			37.85 (9.73)		
III	50	37.60	48.52 (10.81)			34.14 (6.42)		
Types of residency training program								
Professional degree postgraduates	65	48.87	48.31 (8.46)	t = 1.59	0.11	36.34 (7.18)	t = 0.05	0.95
Non-professional degree postgraduates	68	51.13	44.89 (11.54)			36.25 (9.15)		
Family residence before enrollment								
City	52	39.09	46.03 (10.56)	F = 1.22	0.29	34.78 (7.49)	F = 3.03	0.05
County	65	48.87	48.69 (8.56)			36.66 (7.62)		
Countryside	16	12.04	48.31 (7.86)			40.06 (2.02)		
Prefer to work in radiology department								
Yes	121	90.98	47.80 (9.45)	t = -0.75	0.45	36.26 (7.94)	t = 0.35	0.73
No	12	9.02	45.66 (8.26)			37.08 (5.55)		
Personality type								
Introverted personality	56	42.11	47.49 (7.75)	t = 0.17	0.87	36.38 (7.68)	t = -0.09	0.93
Extroverted personality	77	57.89	47.77 (11.25)			36.26 (7.90)		
Family cares for you								
Yes	126	94.74	47.64 (9.45)	t = -0.24	0.81	35.75 (3.30)	t = -0.15	0.87
No	7	5.26	46.50 (5.19)			36.35 (7.85)		
Residency instructor/faculty care for you								
Yes	123	92.48	47.79 (9.34)	t = -0.81	0.42	36.04 (7.74)	t = 1.56	0.12
No	10	7.52	45.30 (9.53)			40.00 (7.30)		
Have you received any medical ethics and humanities training previously in medical school?								
Yes	98	73.68	49.37 (7.98)	t = 3.25	0.00*	35.81 (7.44)	t = 0.53	0.58
No	35	26.32	43.93 (10.90)			36.58 (7.92)		
Have you received any CSs training previously in medical school?								
Yes	78	58.65	48.08 (8.92)	t = 3.47	0.00*	36.08 (7.62)	t = -2.17	0.03*
No	55	41.35	32.25 (11.17)			44.50 (8.66)		
The doctor’s attire can function as an effective tool of non-verbal communication to establish a good patient-doctor relationship								
Agree	90	67.67	48.08 (9.06)	t = 2.51	0.01*	36.30 (7.73)	t = -0.23	0.82
Disagree	43	32.33	39.14 (10.97)			37.00 (8.67)		
Have you ever experienced difficult conversation?								
Yes	97	72.93	46.55 (9.89)	t = -0.79	0.43	38.09 (7.45)	t = -4.60	0.00*
No	36	27.07	48.00 (9.16)			31.61 (6.52)		
How would you rate the current doctor–patient relationship in China in general?								
Tense doctor–patient relationship	59	44.36	45.79 (9.13)	F = 0.88	0.41	37.27 (8.06)	F = 1.87	0.15
Neutral	54	40.60	48.52 (9.36)			36.03 (7.75)		
Harmonious doctor–patient relationship	20	15.04	49.80 (8.56)			34.70 (6.46)		
Personal CSs level								
Good	42	31.58	49.72 (5.15)	F = 0.77	0.46	34.23 (5.83)	F = 5.86	0.00*
Neutral	18	13.54	46.47 (10.10)			33.17 (7.81)		
Poor	73	54.88	47.74 (9.68)			38.33 (8.20)		

Note: The values are given as mean (standard deviation, SD)

Abbreviations: *PAS* positive attitude score, *NAS* negative attitude score, *CS* communication skills, *F* one-way analysis of variance test, *t* independent samples *t*-test

*Statistically significant

### Analysis of CSAS score and factors affecting residents’ PAS and NAS

As shown in Table 1, the mean total scores for the two dimensions of the CSAS were 47.61 ± 9.35 in the PAS and 36.34 ± 7.75 in the NAS. PAS scores were significantly higher for residents who had previously received any CS or medical ethics and humanities training in medical school, in comparison with residents who had never received any training in CS (*P* < 0.01) or medical ethics and humanities (*P* < 0.01). Compared with residents who disagreed that the doctor’s attire can function as an effective tool of non-verbal communication to establish a good patient–doctor relationship, those who agreed had a significantly higher PAS score (*P* = 0.01).

Univariate analysis of NAS scores showed statistically significant differences among residents who had experienced any difficult conversations, different resident grades, those who had received CS training, and personal CS level, indicating that these four items influenced negative attitudes of residents toward learning DPC skills (all *P* < 0.05). Subgroup analyses in subsequent sections were performed for resident grade and personal CS level. Compared with residents in the third year of training, those in the first year had significantly higher NAS scores (*t* = − 3.38, *P* = 0.04). There was no significant difference in the NAS scores between first- and second-year residents (*t* = − 0.34, *P* = 0.99) nor between second- and third-year residents (*t* = 3.72, *P* = 0.15). NAS scores were significantly higher for residents with poor personal CS when compared with those who had good CS skills (*t* = − 4.09, *P* = 0.00); there was no significant difference in NAS scores between residents with neutral and poor personal CS (*t* = 5.16, *P* = 0.06) nor between residents with neutral and good personal CS levels (*t* = − 1.07, *P* = 0.94).

### Residents’ knowledge, experience, and confidence regarding DPC in radiology

**Fig. 1:**
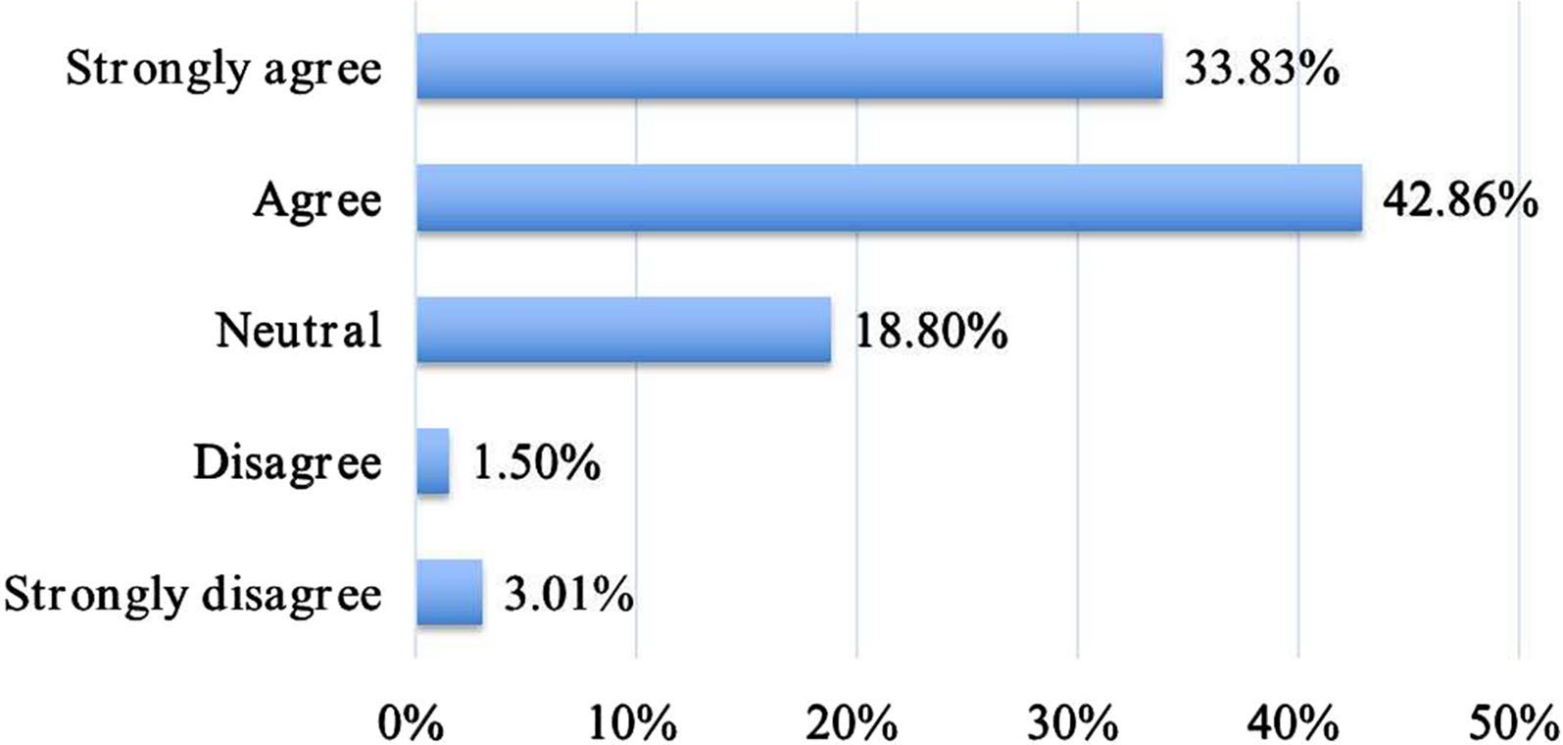
Graph showing respondents’ overall perception of the impact of DPC failure. DPC, doctor–patient communication

As shown in Table 1, only 15.04% (20/133) of residents rated current doctor–patient relationships in China as generally harmonious; 40.60% (54/133) rated these as neutral and 44.36% (59/133) rated doctor–patient relationships as tense. A total of 90/133 (67.67%) agreed that the doctor’s attire can function as an effective non-verbal communication tool to establish a good doctor–patient relationship. Most respondents (97/133; 72.93%) reported having a failed CS experience in at least one difficult conversation in their medical careers. Three-quarters (112/133) of respondents said they “strongly agree” or “agree” that failure in communication has a negative effect on clinical work (Fig. 1).

**Fig. 2:**
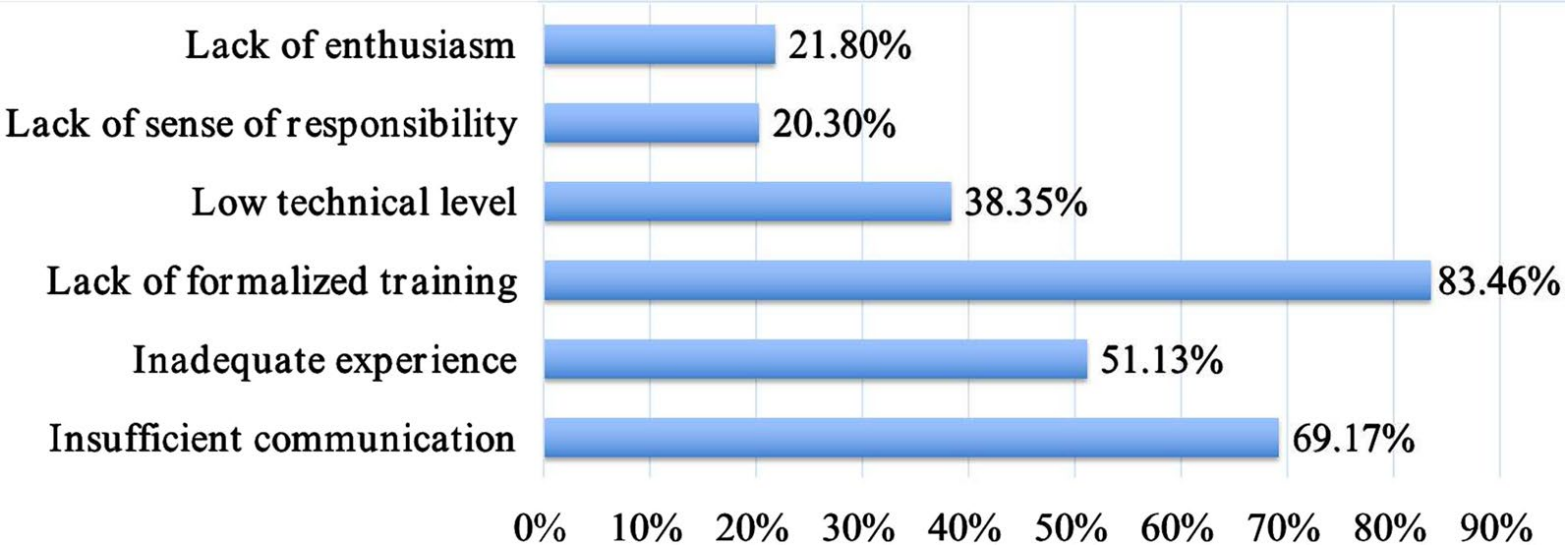
Main reported factors associated with the doctor in the case of DPC failure. DPC, doctor–patient communication

**Fig. 3:**
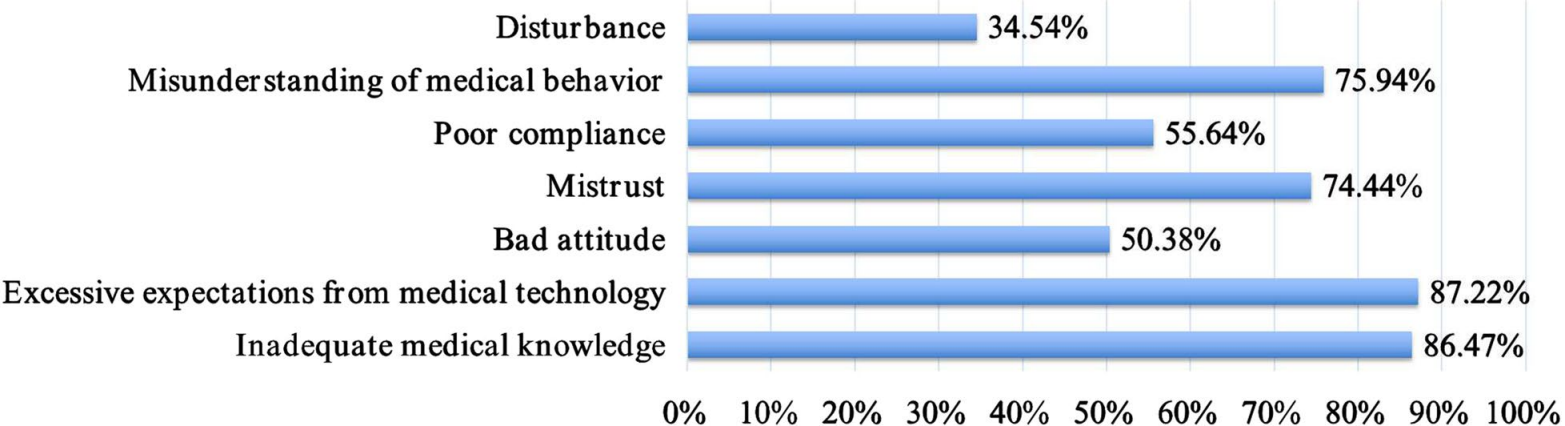
Main reported factors associated with the patient in the case of DPC failure. DPC, doctor–patient communication

The main reported doctor-related factors leading to failure communication are shown in Fig. 2; these factors are “lack of formalized training” (111/133), “insufficient communication” (92/133), and “inadequate experience” (68/133). The most commonly reported patient-related factors that influence DPC were “excessive expectations of medical technology” (116/133), “inadequate medical knowledge” (115/133), “mistrust” (99/133), and “misunderstanding of medical behavior” (101/133); see Fig. 3.

**Fig. 4:**
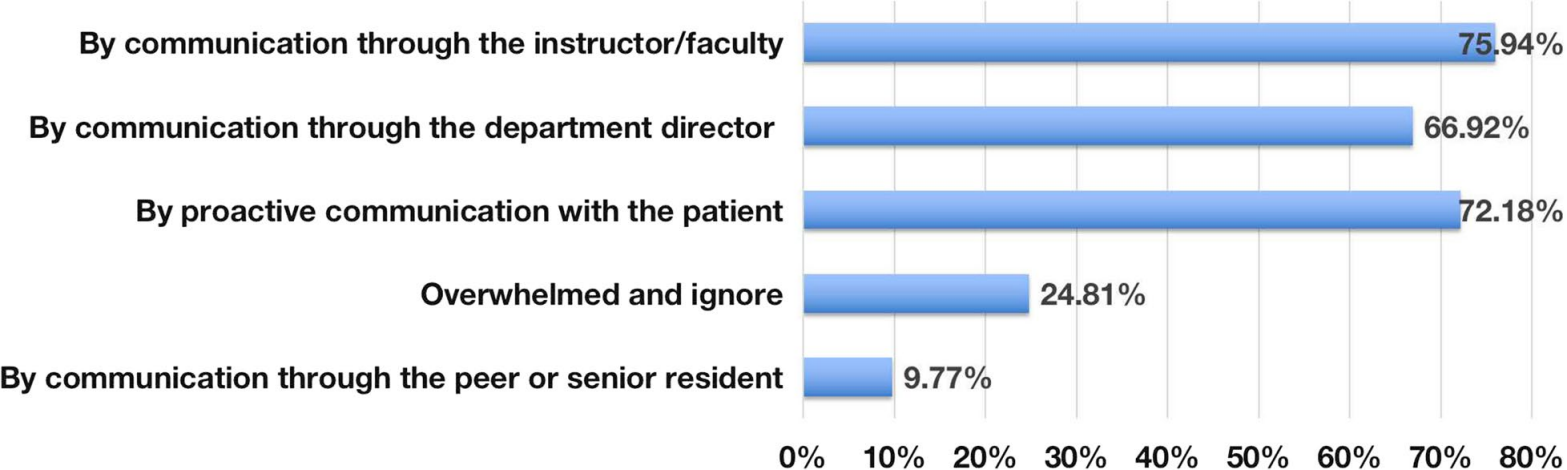
Main reported ways respondents dealt with a difficult conversation

Regarding the multiple-choice question of how to deal with difficult conservations, the most frequent responses were through proactive communication with the patient (96/133), communication through the instructor/faculty (101/133), communication through the department director (89/133), communication through a peer or senior resident (13/133), and feeling overwhelmed and ignoring the patient (33/133) (Fig. 4).

### Interest in receiving formal training in CS and barriers to implementation

Most residents (117/133) said that they “agree” or “strongly agree” that CS can be taught via courses and that receiving formal training in difficult conversations was important for their careers. Ninety-four respondents (70.67%) showed an interest in formalized CS courses. When asked “How often do you expect the course to run?”, 65/133 (48.87%) residents said once a quarter. Surprisingly, only 11/133 (8.27%) respondents chose once every 2 weeks. A lack of time, lack of a standardized curriculum, and lack of educational materials were the most commonly cited barriers to formalized training. Another important barrier was a lack of faculty expertise. All responses are summarized in Table 2.

**Table 2:** Interest in receiving formal training in CS and barriers to implementation (n = 133)

	N	%
CS can be taught via courses		
Strongly disagree	3	2.26
Disagree	1	0.75
Neutral	12	9.02
Agree	71	53.38
Strongly agree	46	34.59
Your interest level of CS training		
Strongly not interest	4	3.01
No interest	2	1.5
Neutral	33	24.81
Interest	60	45.11
Strongly interest	34	25.56
Frequency of training courses you expect		
Once a week	18	13.53
Once every two weeks	11	8.27
Once a month	39	29.32
Once a quarter	65	48.87
Major barriers in implementing formal training		
Heavy work stress or lack of time	71	53.38
Lack of enthusiasm	46	34.59
Lack of educational materials	78	58.65
Lack of faculty expertise	55	41.35
Lack of standardized curriculum	92	69.17

Abbreviation: *CS* communication skills

## Discussion

DPC training programs are a very important part of postgraduate training to become a qualified doctor [20]. Communication is one of the core competencies of radiology residents [21]. The provision of effective and comprehensive DPC training programs for residents is substantially lacking in China. Moreover, the educational curriculum focuses solely on theory [16]. To our knowledge, this study is the first to explore radiology residents’ perceptions, attitudes, and factors regarding DPC and to further investigate learners’ needs with respect to DPC training, with specific goals that can lead to future in-depth research and help guide curriculum planning.

Our study findings indicated that radiology residents’ general attitudes toward receiving CS training were positive, as indicated by high PAS scores accompanied by the strong belief that CS could be taught. As expected with such a positive mindset, radiology residents showed a high willingness to improve their CS through a standardized training curriculum [22]. We identified that the main factor influencing PAS scores regarding the doctor–patient relationship was having any previous training in CS or medical ethics and humanities. In accordance with earlier studies, nearly 42% of radiology residents in our study reported not receiving any CS training and most did not have enough confidence to independently manage difficult conversations [22–24]. These results demonstrate that China’s current post-graduate formal training in DPC skills is suboptimal. A study conducted by Zhou et al. showed that neurology residents who received prior CS training had more confidence and less stress when they encountered difficult conversations, in comparison with those with no previous training [24]. These studies have demonstrated that CS training programs can improve the communication competency of residents, which could be a helpful approach to avoiding or solving conflicts and rebuilding patient–physician trust [24–26]. Interestingly, previous research has shown that the doctor’s attire can function as an effective non-verbal communication tool that signals confidence, trust, and empathy and can help to establish a good patient–doctor relationship [17]. Our study also revealed that the doctor’s white coat and formal clothes was a factor positively associated with the PAS score.

Personal factors, such as one’s knowledge and experience, increase confidence and self-esteem and subsequently have an impact on interprofessional communication [12]. Many residents perceived barriers to interprofessional communication when explicitly expressing a lack of knowledge whereas others indicated that being open about a knowledge gap enabled communication, rather than acting as a barrier [26]. Univariate analysis of NAS scores in our study revealed statistically significant differences among residents who had experienced any difficult conversations, had different resident grades, had previous CS training, and personal CS levels. Subgroup analyses indicated that being in the first year of training was associated with higher NAS scores, and the year of training had a direct negative effect on NAS scores. This may be because junior residents tend to be less experienced and lack expertise compared with senior residents [12].

Prior research has shown that the perception of a harmonious relationship with patients is positively associated with a preference for patient-focused clinical communication [22]. Unfortunately, the relationship between clinicians and patients is still a concern in China. Failure to communicate is one of the main reasons related to doctor–patient tension and loss of trust [25]. In some cases, this has led to verbal and physical violence against medical staff in recent years [27, 28]. The reasons for these behaviors are manifold, but the communication barrier plays a major role. In the present study, only 15.04% (20/133) of residents rated current doctor–patient relationships in China in general as harmonious, as reported in previous studies. Most respondents (97/133; 72.93%) reported having failed in at least one difficult conversation in their medical career. Research has indicated that communication problems are caused by both sides in the doctor–patient relationship [25, 27, 28]. For doctors, the main related factors include professional title, failure to diagnose and treat, misdiagnosis and mistreatment, delayed diagnosis and treatment, poor surgery, poor assessment of condition, low technical level, lack of experience, and poor case recording. For patients, misunderstanding of medical behavior and high prognosis expectations predominate. Surprisingly, ‘patient with inadequate medical knowledge’ is also reported by our participants as a factor associated with failed DPC. After all, this item seems unfair to patients as they should not be expected to have inadequate medical knowledge. The reason for this is probably due to the inexperience of the residents and the mistrust between the young doctors and the patients. Therefore, to bridge the gap between residents and patients created by the nature of professional medicine, improving residents’ CS is critical.

It is important to understand the barriers a radiology resident perceives in practicing good CSs. In the present study, we found barriers to the development and implementation of training in how to handle difficult conversations, such as a lack of a standardized curriculum, lack of time, and lack of enthusiasm, similar to previous reports in other medical and surgical specialties [22, 24, 26, 29]. Another important barrier was faculty expertise. Recently, Bai et al. designed a modified DPC training program for surgical residents in China and reported improved DPC competency among surgical residents, increased satisfaction levels among both standardized patients and surgical residents, and improved consistency of evaluation between standardized patients and surgical residents during doctor–patient encounters [30]. Therefore, hospital administrators and instructional managers should consider developing communication curricula in radiology residencies aimed at equipping faculty with the skills to effectively teach and assess CS. The following key elements should be considered when developing a successful CS curriculum. (1) Curriculum topics: handling conflict, breaking bad news, error disclosure, end-of-life care, patient handoffs, informed consent, and so on; (2) Teaching and assessment methods: didactic lecture, role play, simulation with standardized patients, small group discussion, faculty observation with feedback; (3) Other factors warrant special attention to ensure the delivery of the curricula, including more time for residents to participate in the curriculum by reducing clinical workload, faculty expertise, equipment, administrative assistance, and communication cultural backgrounds.

A recent study found that the number of CS training courses offered by institutions in China remains low, with tertiary hospitals organizing more courses than secondary/primary hospitals [22]. They also found that working at a primary or secondary hospital, and lower positive attitudes toward CS were determined as risk factors for verbal attacks. Our results confirmed the low prevalence of CS training among most Chinese physicians, although, as previously reported, they were very positive about having received CS training [22]. Thus, more efforts and investments are essential to provide CS training to physicians at hospitals, especially on the primary and secondary levels.

This study had several limitations. First, this was a cross-sectional observational study; therefore, no causal relationships can be assumed. It is also important to note the short survey period. The attitudes of medical professionals may change over time. Second, the participants in this study were drawn from a sample in nine qualified programs in the province of Guangdong, which limits the external validity of the study and generalizability of the findings. Further studies are needed using a more representative, larger sample, which should include a comparative study across different regions and settings. Third, the results of this survey may have been influenced by the different backgrounds of the residents. Fourth, some of the questions in this survey lack a clearer description of residents’ attitudes and experiences in the DPC, and residents reporting their perceptions using only a binary response scale for the scale, such as for the question about difficult conversations, may bias the study results. For further studies, more precise questions that accurately reflect the DPC and the combination of qualitative methods, such as focus groups, could provide an in-depth understanding of residents’ opinions on the DPC. Furthermore, our survey only focused on residents. Program directors and patients were not included, which may have introduced reporting bias. Future studies that include program directors and patient evaluations are needed to inform curriculum development.

## Conclusion

This study provides a preliminary assessment of the current status of training in DPC in nine qualified radiology residency programs in China. Overall, the survey showed that radiology residents have received little training in communicating with patients. Despite this, most residents expressed interest in receiving additional training in DPC skills. Time and lack of a standardized curriculum, faculty expertise, and materials were the most common barriers to formalized training. Establishing and promoting a targeted communication curriculum for Chinese radiology residents should be a future focus.

### Supplementary Information

**Additional file 1.** Supplementary material.

## Data Availability

The datasets used and/or analyzed during the current study are available from the corresponding author upon reasonable request.

## Abbreviations

DPC: Doctor–patient communication
CS: Communication skills
SRT: Standardized residency training
CSAS: Communication Skills Attitude Scale
PAS: Positive attitude scale
NAS: Negative attitude scale
