# Supplementary material for: Attitudes toward communication skills with learner needs assessment within radiology residency programs in China: a cross-sectional survey
Source: BMC Res Notes. 2024 Apr 23;17:114. doi: 10.1186/s13104-024-06779-8 (PMC11036608; doi:10.1186/s13104-024-06779-8)
Supplement: Supplementary file 1 — Additional file 1. Supplementary material. [file 13104_2024_6779_MOESM1_ESM.docx]

Needs assessment questionnaire on doctor–patient communication skills in a radiology residency training program

This questionnaire is designed to evaluate the status of radiology residency training in terms of doctor–patient communication skills. We guarantee that all collected information will be used for research purposes only. Your personal information will be kept confidential. Thank you very much for taking time from your busy schedule to complete our questionnaire. We hope that this questionnaire will be beneficial for all residents.

If you have any question, please feel free to talk to staff distributing the questionnaire.

# Part I: Demographic details

| What is your sex? | □ Male □ Female |
| --- | --- |
| Age | __________ years old. |
| What is your highest level of medical education? | □ Junior college □ Bachelor’s  □ Master’s □ Doctorate |
| In which hospital are you currently working? |  |
| Marital status | □ Single/divorced □ Married |
| What’s your seniority? | □ PGY 1 □ PGY 2 □ PGY 3 |
| What’s your type of residency training program? | □ Professional degree postgraduate  □ Non-professional degree postgraduate |
| Where is your native place of residence? | □ City □ County □ Countryside |
| Would you like to work in radiology？ | □ Yes  □ No |
| What type of personality do you have？ | □ Introverted personality  □ Extroverted personality |
| Do you feel your family cares about you？ | □ Yes  □ No |
| Do you feel your instructor/faculty cares about you? | □ Yes  □ No |

PGY, postgraduate year.

**Part II Residents’ knowledge, experience, and confidence regarding doctor–patient communication in radiology**

Please indicate the degree of agreement with the following questions and statements by choosing the most appropriate response on the given scale of 1 to 5.

| **1** | **2** | **3** | **4** | **5** |
| --- | --- | --- | --- | --- |
| **Strongly disagree** | **Disagree** | **Neutral** | **Agree** | **Strongly agree** |

| How would you rate current doctor–patient relationships in China in general?  □ Tense  □ Neutral  □ Harmonious | | | | | |
| --- | --- | --- | --- | --- | --- |
| Have you previously received any communication skills training in medical school?  □ Yes  □ No | | | | | |
| Have you previously received any medical ethics and humanities training in medical school?  □ Yes  □ No | | | | | |
| The doctor’s attire (white coat and formal clothes) can function as an effective non-verbal communication tool to establish a good patient–doctor relationship.  □ Agree  □ Disagree | | | | | |
| Have you ever experienced difficult conversations (e.g., breaking bad news, disclosing medical errors, etc.)?  □ Yes  □ No | | | | | |
| How would you rate your own personal communication skills level?  □ Good  □ Neutral  □ Poor | | | | | |
| Please choose the doctor-related factors that you believe lead to failed communication:__________ (multiple answers possible)   1. Insufficient communication 2. Inadequate experience 3. Lack of formalized training 4. Low technical level 5. Lack of sense of responsibility 6. Lack of enthusiasm | | | | | |
| Please choose the patient factors that you believe lead to failed communication:__________ (multiple answers possible)   1. Inadequate medical knowledge 2. Excessive expectations of medical technology 3. Poor attitude 4. Mistrust 5. Poor compliance 6. Misunderstanding of medical behavior | | | | | |
| How do you deal with difficult conservations: _______? (multiple answers)   1. By communication through a peer or senior resident 2. Feeling overwhelmed and ignoring the patient 3. Through proactive communication with the patient 4. Through communication through the department director 5. Through communication through the instructor/faculty | | | | | |
| Failure in doctor–patient communication has a negative effect on clinical work. | 1 | 2 | 3 | 4 | 5 |

**Part III Communication Skills Attitude Scale**

Please indicate the degree of agreement with the following questions and statements by choosing the most appropriate response on the given scale of 1 to 5.

| **1** | **2** | **3** | **4** | **5** |
| --- | --- | --- | --- | --- |
| **Strongly disagree** | **Disagree** | **Neutral** | **Agree** | **Strongly agree** |

| **No.** | **Item** | **PAS/NAS*** | **Agreement scale** | | | | |
| --- | --- | --- | --- | --- | --- | --- | --- |
| 1. | To be a good doctor, I must have good communication skills. | PAS | 1 | 2 | 3 | 4 | 5 |
| 2. | I cannot see the point of learning communication skills. | NAS | 1 | 2 | 3 | 4 | 5 |
| 3. | Nobody is going to fail their medical degree because of having poor communication skills. | NAS | 1 | 2 | 3 | 4 | 5 |
| 4. | Developing my communication skills is just as important as developing my knowledge of medicine. | PAS | 1 | 2 | 3 | 4 | 5 |
| 5. | Learning communication skills has helped or will help me respect patients. | PAS | 1 | 2 | 3 | 4 | 5 |
| 6. | I do not have time to learn communication skills. | NAS | 1 | 2 | 3 | 4 | 5 |
| 7. | Learning communication skills is interesting. | PAS | 1 | 2 | 3 | 4 | 5 |
| 8. | I cannot be bothered to turn up to sessions on communication skills. | NAS | 1 | 2 | 3 | 4 | 5 |
| 9. | Learning communication skills has helped or will help facilitate my teamwork skills. | PAS | 1 | 2 | 3 | 4 | 5 |
| 10. | Learning communication skills has improved my ability to communicate with patients. | PAS | 1 | 2 | 3 | 4 | 5 |
| 11. | Communication skills training states the obvious and then complicates it. | NAS | 1 | 2 | 3 | 4 | 5 |
| 12. | Learning communication skills is fun. | PAS | 1 | 2 | 3 | 4 | 5 |
| 13. | Learning communication skills is too easy. | NAS | 1 | 2 | 3 | 4 | 5 |
| 14. | Learning communication skills has helped or will help me respect my colleagues. | PAS | 1 | 2 | 3 | 4 | 5 |
| 15. | I find it difficult to trust information about communication skills given to me by non-clinical lecturers. | NAS | 1 | 2 | 3 | 4 | 5 |
| 16. | Learning communication skills has helped or will help me recognize patients' rights regarding confidentiality and informed consent. | PAS | 1 | 2 | 3 | 4 | 5 |
| 17. | Communication skills training would have a better image if it sounded more like a scientific subject. | PAS | 1 | 2 | 3 | 4 | 5 |
| 18. | When applying for medical school, I thought it was a really good idea to learn communication skills. | PAS | 1 | 2 | 3 | 4 | 5 |
| 19. | I do not need good communication skills to be a doctor. | NAS | 1 | 2 | 3 | 4 | 5 |
| 20. | I find it hard to admit to having some problems with my communication skills. | NAS | 1 | 2 | 3 | 4 | 5 |
| 21. | I think it is really useful to learn communication skills as part of the medical degree. | PAS | 1 | 2 | 3 | 4 | 5 |
| 22. | My ability to pass exams will get me through medical school rather than my ability to communicate. | NAS | 1 | 2 | 3 | 4 | 5 |
| 23. | Learning communication skills is applicable to learning medicine. | PAS | 1 | 2 | 3 | 4 | 5 |
| 24. | I find it difficult to take learning communication skills seriously. | NAS | 1 | 2 | 3 | 4 | 5 |
| 25. | Learning communication skills is important because improving my ability to communicate is a lifelong skill. | PAS | 1 | 2 | 3 | 4 | 5 |
| 26. | Learning communication skills should be left to psychology students, not medical students. | NAS | 1 | 2 | 3 | 4 | 5 |

*PAS, positive attitude scale; NAS, negative attitude scale.

**Part IV Residents’ interest in receiving formal training in doctor–patient communication skills and barriers to implementation**

By choosing the most appropriate response on the given scale of 1 to 5, please indicate the degree of agreement with the following questions and statements.

| **Strong disinterest** | **No interest** | **Neutral** | **Interest** | **Strong interest** |
| --- | --- | --- | --- | --- |
| **1** | **2** | **3** | **4** | **5** |
| **Strongly disagree** | **Disagree** | **Neutral** | **Agree** | **Strongly agree** |

| Doctor–patient communication skills can be taught via courses. | 1 | 2 | 3 | 4 | 5 |
| --- | --- | --- | --- | --- | --- |
| Your interest level of doctor–patient communication skills training | 1 | 2 | 3 | 4 | 5 |
| In your opinion, what’s the frequency of training courses you expect?  □ Once a week  □ Once every 2 weeks  □ Once a month  □ Once a quarter | | | | | |
| In your opinion, please choose the barriers to implementing formal training: ______(multiple answer possible)   1. Heavy work stress or lack of time 2. Lack of enthusiasm 3. Lack of educational materials 4. Lack of faculty expertise 5. Lack of standardized curriculum | | | | | |
